# Loss of CpG island immunity to DNA methylation induced by mutation

**DOI:** 10.1186/s13072-023-00488-5

**Published:** 2023-05-11

**Authors:** Bernhard Horsthemke, Adrian Bird

**Affiliations:** 1 Institut Für Humangenetik, Universitätsklinikum Essen, Universität Duisburg-Essen, 45122 Essen, Germany; 2 Wellcome Centre for Cell Biology, University of Edinburgh, Edinburgh, EH9 3BF UK

**Keywords:** Epigenetics, DNA methylation, CpG island, Transgenerational inheritance, Acquired characters, Mutation

## Abstract

The inheritance of acquired traits in mammals is a highly controversial topic in biology. Recently, Takahashi et al. (Cell 186:715–731, 2023) have reported that insertion of CpG-free DNA into a CpG island (CGI) can induce DNA methylation of the CGI and that this aberrant methylation pattern can be transmitted across generations, even after removal of the foreign DNA. These results were interpreted as evidence for transgenerational inheritance of acquired DNA methylation patterns. Here, we discuss several interpretational issues raised by this study and consider alternative explanations.

## Background

The possibility that acquired human characteristics can be inherited across generations has held an enduring fascination over centuries, despite controversial evidence [2, 3]. In the latest phase of this on-going debate, attention has focused particularly on DNA methylation, whose pattern tends to be copied when DNA replicates [4]. This potential for maintenance despite the disruption caused by synthesis of new DNA has made cytosine methylation in the self-complementary sequence CpG the medium of choice when postulating transfer of epigenetic information between generations. The recently published paper by Takahashi et al. [1] follows this tradition by tracking DNA methylation at two artificially methylated CpG islands (CGIs) between generations in mice. Strikingly, this post-synthetic modification of DNA, once imposed, appears to be transmitted across multiple mouse generations. Here, we highlight several interpretational issues raised by this interesting study.

## A transient mutation remembered by the epigenome?

To induce de novo methylation at hitherto unmethylated CGIs, the authors inserted a stretch of foreign CpG-free DNA into the embryonic stem cell genome close to either of the two promoters. The use of the word “acquired” to describe the imposition of the methylated state at these loci recalls the Lamarckian concept of inheritance of acquired characteristics, but, unlike adaptations caused by behavior or the environment, this one is triggered by drastic genetic manipulation. Thus, although the authors provocatively chose to target two genes (*Ankrd26* and *Ldlr*) that have been implicated in epigenetic transmission of metabolic phenotypes between generations [5], the initiating event in this case is not the environment, but a mutation. It is nevertheless remarkable that when the inserted DNA was removed, leaving a small genetic scar (TTAA instead of TTCT and GTAC, respectively), the methylated state appeared to persist, not only in cultured cells but across many generations of mice derived from these cells.

While DNA methylation is the key “phenotype” being inherited, several lines of evidence suggest that it is not bearer of this memory. Firstly, primordial germ cells essentially erase DNA methylation at these CGIs, only restoring it later in development. Secondly, the authors refer to unpublished data showing that artificial methylation of a CGI in embryonic stem cells (ESCs) via recruitment of a DNA methyltransferase (epigenome editing) is not maintained in the same way as their genetic modification approach. Thirdly, as the aberrant methylation is mosaic (i.e., present in a subset of cells only), it is not clear how a mosaic methylation pattern would be inherited, given that a specific parental sperm or egg can only be methylated or be unmethylated. In some mouse lines, the targeted CGIs are even unmethylated in all germ cells and at the blastocyst stage. Fourthly, a purely epigenetic effect would be expected to weaken and fade away after a few generations, whereas levels of DNA methylation at these CGIs remain constant.

Whatever is being memorized at the locus, it does not appear to be DNA methylation. One obvious possibility is that a persisting genetic change (TTAA) renders these loci susceptible to de novo methylation. The authors are at pains to address this objection, as there is extensive evidence that DNA methylation patterns are often affected by DNA sequence polymorphism, pointing to a genetic rather than an epigenetic effect [6]. However, for *Ankrd26*, the authors subsequently derived scar-free cell and mouse lines by targeting a TTAA site 45 bp upstream of the TTCT site and here again the methylated state of the *Ankrd26* SL allele was observed in three generations. Evidently, the presence of the scars did not guarantee the methylated state as the allele reverted to the non-methylated state in some mouse lines. Moreover, extensive DNA sequencing in the surrounding genomic region detected no other legacy DNA sequence changes.

If neither DNA methylation nor genome sequence were responsible, what are the alternatives? There is a precedent where naturally acquired DNA methylation survives the global demethylation that accompanies early mammalian development (although the modification is erased prior to the next generation). This concerns imprinted genes, where the methylated alleles specifically recruit KRAB zinc finger proteins which, with the aid of the repressor KAP1, protect the methylated allele [7]. Persistent methylation of transposable elements can also involve KRAB zinc finger proteins, but in this case DNA methylation is transiently lost during differentiation of the germline and subsequently restored as development proceeds [8]. In both these cases, continuity of DNA methylation depends upon DNA sequence-specific repressor proteins. These mechanisms could only explain consistent re-establishment of CGI methylation as reported by Takahashi and colleagues if the newly methylated state itself inadvertently created a DNA binding motif for repressors of this kind. In this context, it may be of interest that methylation of the *Ankrd26* CGI gives rise to a binding motif for ZFP57 (TGCmCGC), a mouse protein implicated in protecting methylated imprinted loci against loss of methylation during early development [9, 10]. Also a new ZFP57 binding site is formed close to the *Ldlr* CGI. Neither of these identical sequence motifs would be capable of recruiting ZFP57 in the absence of CG methylation, but whether a single site could contribute to sustained methylation of these entire CGIs is unknown.

An interesting alternative mechanism depends on evidence that transcriptional activity or silence drives DNA methylation states. In this regard, it is important to note that the vast majority of vertebrate genomic DNA is highly methylated at CpG sites. Not only is heterochromatin methylated, but also to a roughly equivalent extent euchromatin, including intergenic DNA and gene bodies. So while is often assumed that DNA methyltransferases (DNMTs) are actively recruited to discrete target loci, the ubiquity of CpG methylation resembles a default state. Evidence that chromatin modifications can influence de novo DNMT recruitment (e.g., histone H3K36me2 [11]) appears to contradict this assertion, but these interactions arguably modulate rather than switch local DNMT activity. It is possible that the primary determinant of whether a CpG becomes methylated may not be attraction, but repulsion of DNMTs. In other words, DNA methylation goes everywhere that it is not excluded, most often by chromatin that has been or still is involved in active transcription, such as CGIs [12]. Mechanistically, we know that H3K4me3—a characteristic histone mark of CGIs and active promoters—excludes DNMT3A and DNMT3B from chromatin (reviewed in [13]). It appears that insertional mutagenesis has permanently crippled the ability of the affected CGIs to exclude DNA methylation, perhaps due to irrevocable interference with promoter function and concomitant alteration of the histone modification landscape. If the methylation-free status of each CGI indeed depends on active transcription at critical stages of development, it is possible that the high levels of DNA methylation following insertional mutagenesis may deny access to one or more transcription factors. Conceivably, this may tip the balance in favor of locus silencing at critical developmental stages, leading to absence of H3K4me3 and effectively permanent DNA methylation.

## Concluding remarks

In summary, the interesting study of Takahashi et al. [1] shows that the privileged immunity to DNA methylation of a CGI can be seriously compromised by transient local alteration of its DNA sequence. We now need to understand the molecular mechanisms that underlie this abrupt change of epigenetic status. While DNA methylation itself may be a symptom rather than a cause of the transition, other genetic or epigenetic players may be involved. Importantly, the relevance of this model system to acquisition and transmission of naturally occurring epigenetic variation has yet to be established.

## Data Availability

Not applicable.

## Abbreviations

CpG: Cytosine-guanine dinucleotide
CGI: CpG island
DNMT: DNA methyltransferase
ESC: Embryonic stem cell
H3K4me3: Histone 3 lysine 4 trimethylation
H3K36me2: Histone 3 lysine 36 dimethylation
KAP1: KRAB-associated protein 1
KRAB: Krüppel-associated box
ZFP57: Zinc finger protein 57
